# The Role of Elective Neck Treatment in the Management of Sinonasal Carcinomas: A Systematic Review of the Literature and a Meta-Analysis

**DOI:** 10.3390/cancers13081842

**Published:** 2021-04-13

**Authors:** Costanza Galloni, Luca Giovanni Locatello, Chiara Bruno, Angelo Cannavicci, Giandomenico Maggiore, Oreste Gallo

**Affiliations:** 1 Department of Otorhinolaryngology, Careggi University Hospital, Largo Brambilla 3, 50134 Florence, Italy; costanza.galloni@stud.unifi.it (C.G.); chiara.bruno@unifi.it (C.B.); a.cannavicci.md@gmail.com (A.C.); maggiore2@virgilio.it (G.M.); oreste.gallo@unifi.it (O.G.); 2 Department of Experimental and Clinical Medicine, University of Florence, 50134 Florence, Italy

**Keywords:** sinonasal cancer, head and neck cancer, neck dissection, nodal metastasis, radiotherapy

## Abstract

**Simple Summary:**

The impact of elective neck treatment (ENT), whether by irradiation or dissection, on the prognosis of patients with cN0 sinonasal carcinomas remains an understudied issue. A systematic review and meta-analysis of the literature were performed according to PRISMA guidelines in order to assess regional recurrence rates after elective neck treatment compared to observation in cN0 patients. Twenty-six articles for a total of 1178 clinically N0 patients were analyzed. Overall, the 5-year overall survival was 52%; 34.6% of patients underwent elective treatment of the neck and 140 regional recurrences were registered. ENT appears to confer a lower risk of regional recurrence compared to observation alone with a cumulative OR of 0.38 (95% CI 0.25–0.58). Our meta-analysis supports the efficacy of ENT in order to reduce the risk of regional recurrence but the overall impact on survival remains uncertain.

**Abstract:**

The impact of elective neck treatment (ENT), whether by irradiation or dissection, on the prognosis of patients with cN0 sinonasal carcinomas (SNCs) remains an understudied issue. METHODS: A systematic review and meta-analysis of the literature were performed according to PRISMA guidelines in order to assess regional nodal relapse rate after ENT compared to observation in cN0 SNCs patients. Twenty-six articles for a total of 1178 clinically N0 patients were analyzed. Globally, the 5-year overall survival was 52%; 34.6% of patients underwent ENT and 140 regional recurrences were registered (5.9% in the ENT cohort and 15% in the observation group). ENT appears to confer a lower risk of regional recurrence compared to observation alone, with a cumulative OR of 0.38 (95% CI 0.25–0.58). Our meta-analysis supports the efficacy of ENT for reducing the risk of regional recurrence, but its overall impact on survival remains uncertain.

## 1. Introduction

Sinonasal tumors contribute to only 5% of all head and neck cancers, yet they constitute a wide spectrum of pathological entities where malignant epithelial tumors (SNCs, sinonasal carcinomas) represent more than 80% of them [1]. Their management is complex and often requires multimodal approaches that have been shown to improve survival and organ-preservation rates (e.g., orbital contents) compared to surgery alone [2,3,4]. Nonetheless, despite the enormous improvement of endoscopic surgical approaches and radiation delivery techniques, the prognosis remains disappointing, with an overall 5-year survival rate ranging from 30% to 50% [5,6].

Lymphatic drainage from the sinonasal district is mainly directed to the upper jugular, perifacial, and retropharyngeal nodes, while the sentinel lymph node approach is too far to be implemented in SNCs [7,8]. Although the incidence of regional metastasis is notably low at the time of diagnosis (about 10% in a recently published series by Peck et al. [9]), up to 33% of patients will eventually develop them during the follow-up [9,10]. In this regard, the decision whether to adopt a watchful waiting approach or to perform an elective neck treatment (ENT), whether by surgery (END, elective neck dissection) or radiotherapy (ENI, elective neck irradiation), on a clinically node-negative (cN0) SNC remains an understudied issue [11,12]. The present systematic review of the literature and meta-analysis aims to evaluate the prognostic role and clinical relevance of the elective neck treatment in the management of SNCs.

## 2. Materials and Methods

This systematic review and meta-analysis followed the Preferred Reporting Items for Systematic Reviews and Meta-analyses (PRISMA) guidelines [13]. It was registered on the International Prospective Register of Systematic Reviews (PROSPERO, University of York, UK) with the publishing number CRD42020169882.

### 2.1. Searching Methods

We searched Pubmed, Scopus, and Google Scholar from 1980 to December 2020 using the following words, phrases in various combinations, or strings: (“neck dissection” OR “neck metastasis” OR “regional metastasis” OR “regional failure”) AND (“sinonasal malignancies” OR “sinonasal cancer” OR “paranasal cancer” OR “paranasal malignancies”); “sinonasal cancer” AND (regional OR neck OR occult OR failure OR recurrence OR distant OR “neck management”); sinonasal AND “neck dissection”; occult AND (neck OR regional) AND (sinonasal OR paranasal); (paranasal OR sinonasal) AND (occult neck metastas* OR (occult metastas* AND neck)).

Languages of publication were limited to English, French, and Italian. We also searched the reference lists of all relevant systematic reviews and meta-analyses. Two reviewers (CG, LGL) independently screened articles and abstracts recovered by the search. Duplicates were removed using open-source reference management software Zotero Version 5.0.93 for Linux (Corporation for Digital Scholarship, Vienna, VA, USA). Articles deemed potentially relevant were obtained and assessed in detail by each reviewer independently, according to the above criteria. All discrepancies were resolved by consensus.

### 2.2. Eligibility Criteria

Inclusion criteria were as follows: prospective or retrospective studies, randomized controlled trials, and case series; studies including patients with pathologically documented SNCs (squamous cell type, intestinal-type, and non-intestinal adenocarcinoma, sinonasal undifferentiated carcinoma/SNUC, adenoid cystic carcinoma, other malignant salivary gland cancers); studies including detailed clinical data about initial neck status, neck treatment strategy, and subsequent regional recurrences with a minimum follow-up time of 6 months.

Exclusion criteria: studies including patients under 18; studies including other head and neck cancer sites and whose data were not clearly discernible; studies including non-epithelial sinonasal malignancies (mucosal melanoma, olfactory neuroblastoma, lymphomas, sarcomas, etc.); case reports or case series smaller than 5 patients.

### 2.3. Data Collection and Outcome Definitions

The following data were extracted from each study whenever available: demographics (number of patients, age, gender); clinical/surgical data: TNM stage/classification at diagnosis, anatomical subsite involved (maxillary, ethmoidal, frontal or sphenoid sinus, and nasal cavity), the reported final histopathology, treatment modalities (surgical resection irrespective of the endoscopic/open/combined approach chosen), and overall survival (OS). Information was extracted from these cohorts regarding cervical nodal status at presentation, initial treatment for nodal disease: therapeutic neck dissection for N+ cases, no treatment, or elective neck treatment (ENT) that could be both surgical (END, Elective Neck dissection) or by external RT (ENI, Elective Neck Irradiation). Finally, the regional recurrence rate was recorded and, whenever possible, it was noted whether the nodal failure was isolated or concomitant with local or distant relapse. In addition, in both ENT and watchful waiting groups, the chosen salvage neck strategy was retrieved in order to define the impact of the regional failure on prognosis.

The cumulative analysis was performed by including modalities used for treatment, nodal involvement at presentation, or subsequent recurrence. The estimated risk of nodal involvement was calculated by combining nodal involvement at presentation and subsequent recurrence in the neck, whether treated with ENT or no treatment at all.

### 2.4. Quality Assessment

Three reviewers (CG, AC and LGL) independently extracted data from each study, which were reviewed for consistency among the authors, and any discrepancies were resolved by consensus. The risk of bias for each study using the ROBINS tool with any discrepancies was resolved by consensus [14]. Visualization of the risk-of-bias assessments was performed by creating a traffic lights plot and a weighted bar plot using the robvis tool [15].

### 2.5. Statistical Methods

The software RevMan 5.3 (Review Manager, Version 5.3. Copenhagen: The Nordic Cochrane Centre, The Cochrane Collaboration, 2014) was used for the meta-analysis and the creation of the forest plot. The odds ratios (ORs) of regional (neck) nodal failure and their 95% confidence intervals (CIs) were calculated for each study. Statistical heterogeneity was assessed using Cochran’s Q statistic (*p*-value for heterogeneity) and the I^2^ statistic (total percentage of variation resulting from heterogeneity). In the case of significant heterogeneity (I^2^ ≥ 50) the random-effect model was used, while the fixed-effect model was used in absence of significant heterogeneity. Herein, we solely applied the fixed-effect model to obtain ORs, 95% CI, and *p*-values.

## 3. Results

**Figure 1:**
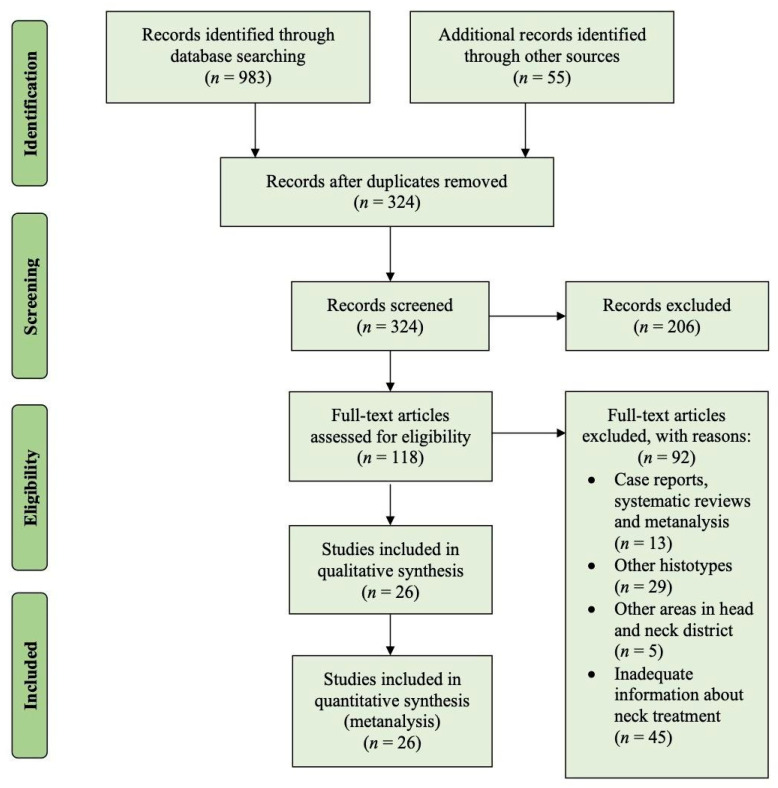
Flow diagram depicting the selection of the papers included in the present review.

Using the selected keywords, we found 983 articles; moreover, we added 55 articles from the reference lists of all the systematic reviews and meta-analyses found in the research. After the removal of duplicates, 324 titles and abstracts were analyzed, and 206 studies were excluded. A total of 118 articles were fully read and a further 92 were excluded because they did not meet the eligibility criteria. In particular, 13 were case reports, systematic reviews, or meta-analyses; 29 included in their analyses or focused only on other non-epithelial histotypes; 5 also included carcinomas of other subsites of the head and neck; and 45 articles did not provide adequate information on neck treatment. In the end, 26 articles were eligible for the meta-analysis, and a PRISMA flow chart is represented in Figure 1.

A descriptive overview of the included articles is presented in Table 1 [16,17,18,19,20,21,22,23,24,25,26,27,28,29,30,31,32,33,34,35,36,37,38,39,40,41]. All the 26 articles included in the meta-analysis were retrospective case series, published from 1987 to 2018. In total, 15 studies (57.7%) were conducted in the USA (6 in Florida, 5 in Texas, 3 in California, and 1 in collaboration between West Virginia and Pennsylvania), 1 was performed in India, 1 in South Korea, 3 in the Netherlands, 2 in the UK, 2 in Germany, 1 in France, and 1 in Spain.

The entire cohort included 1320 patients, with a median of 37 cases for each series. Sex information was not available in 5 studies; in the remaining papers, there were 657 males (64.7%) and 359 females (35.3%). The median age was 58 years, with a range from 8 to 92 years. The most frequent histotype was squamous cell carcinoma (801 cases, 60.7%), followed by adenocarcinoma (190 cases, 14.4%), sinonasal undifferentiated carcinoma (171 cases, 13%), and adenoid cystic carcinoma (120 cases, 9.1%). Regarding the primary subsite, in 339 cases (31.1%) the origin was in the nasal cavity; in 639 cases (63.5%) it was in the maxillary sinus; in 102 (9.3%) it was in the ethmoidal sinus; in 9 cases (0.8%) it was the sphenoidal sinus; and in 2 it was the frontal sinus (0.2%). In 5 studies, the authors did not clearly specify these data. 

In this cohort, 305 carcinomas (29.4%) were T1/T2, 280 (24.2%) were T3, and 570 (49.4%) were T4 (110 T4a, 91 T4b, 369 not specified); 5 studies did not report the T stage. As for the lymph node involvement, only 100 patients (7.5%) had a regional metastasis at diagnosis (N+), against 1198 who were cN0. Of the N+ patients, 49.2% were N1, 50.8% were N2 (N2a in 20% of N2 cases, N2b in 46.7%, and N2c in 33.3%), and none were classified as N3. Exclusive surgical treatment of primary tumor was performed in 101 cases (8.3%), while 393 patients (32.2%) were treated with RT or with RT-CHT. The majority of cases (726 patients, 59.5%) were treated with a combined therapy composed of surgery and adjuvant or neoadjuvant RT/CHT-RT. In one study, the kind of treatment of primary tumor was not reported. Complications after neck irradiation were rarely described: Lee et al. [33] and Hinerman et al. [29], respectively, mentioned a case of left brachial plexopathy with severe neck fibrosis and a carotid blow-out; moreover, Castelnau-Marchand et al. [21] reported 51 cases of dysphagia, 61 of radio-mucositis, and 61 patients who suffered from radiodermatitis related to the neck RT. The mean 2-year and 5-year overall survival rates were 66% and 52%, respectively. The median follow-up time was 54 months, with a range from 10 to 132 months.

**Figure 2:**
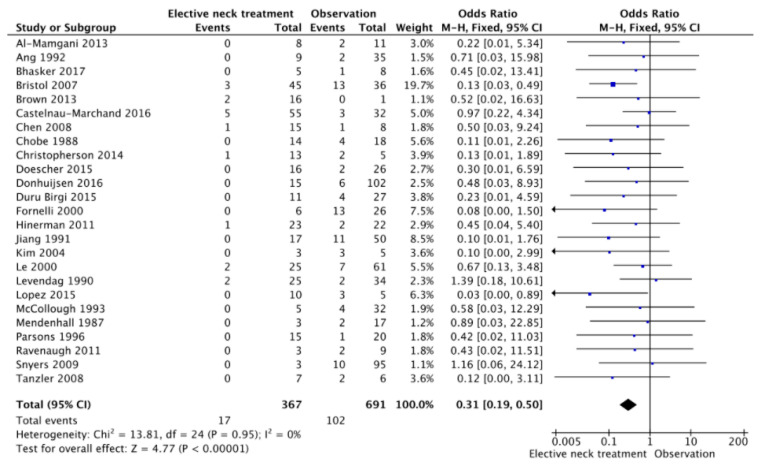
Forest plot of the present meta-analysis.

**Figure 3:**
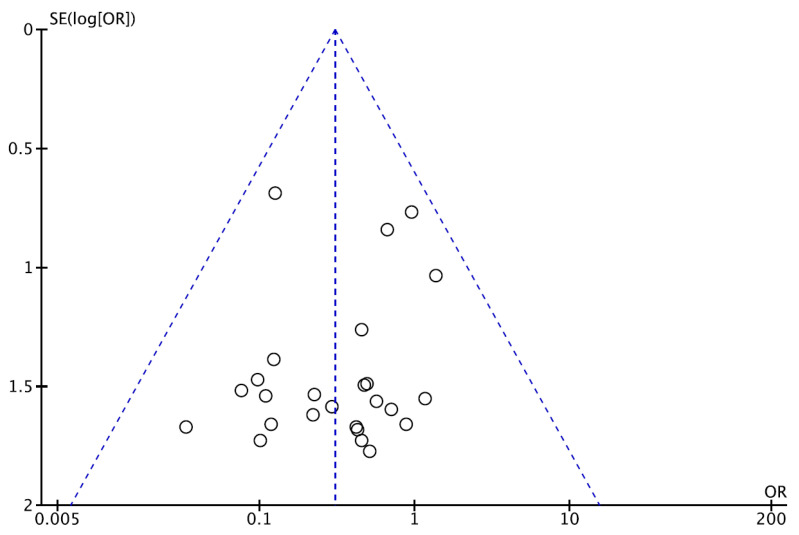
The funnel plot for all the studies included in the present meta-analysis. This is a graphical representation of the size of the papers included, plotted against the effect size they report. Each dot represents a single study; on the y-axis SE (standard error) represents study precision while the x-axis shows the study’s result in terms of odds ratio.

**Figure 4:**
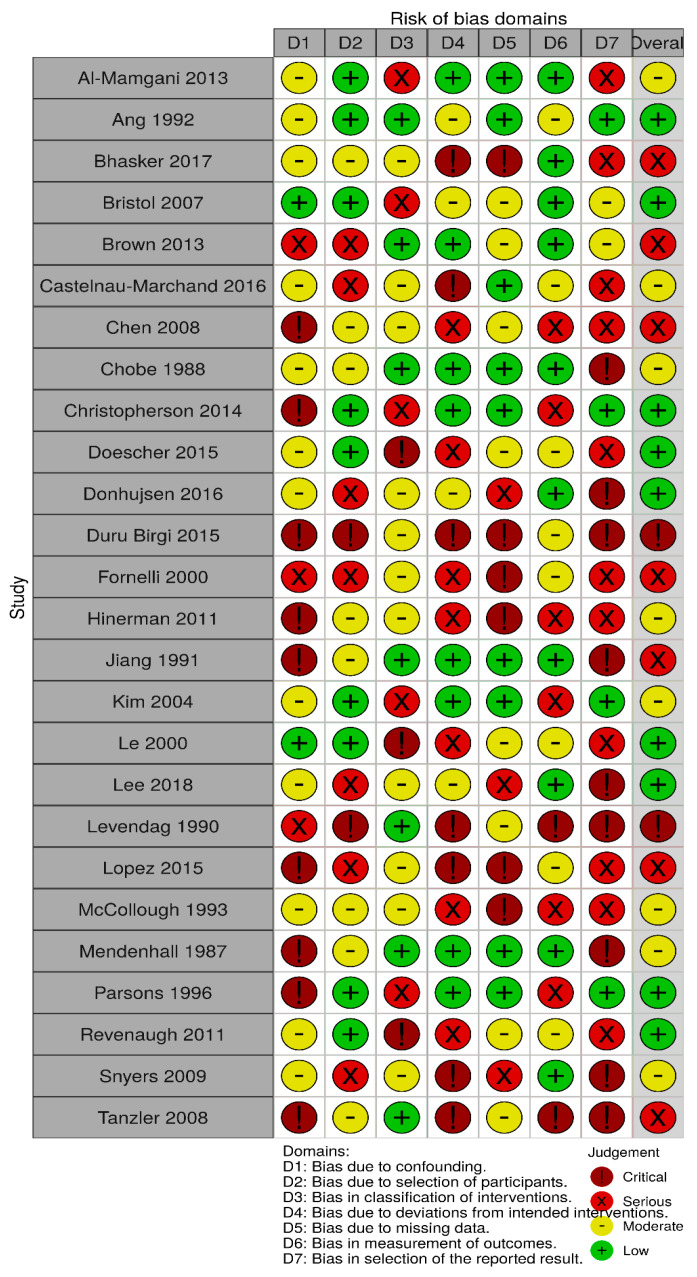
A “traffic light” plot of the domain-level judgements for each individual result.

**Figure 5:**
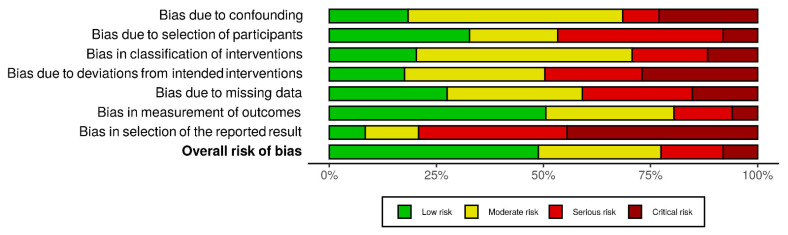
Weighted bar plot of the distribution of risk-of-bias judgements within each bias domain.

Meta-analysis is based on the evaluation of 1178 clinically N0 patients. Of these, 407 (34.6%) underwent ENT while, in 771 cases (65.4%), the neck was not treated, and an “observation” strategy was chosen. In this cohort, 140 nodal failures were observed, with a regional recurrence rate of 5.9% (24/407) in the ENT group and 15% (116/771) in the observation group (Table 2). ORs for regional recurrences after elective neck treatment ranged from a minimum of 0.03 to a maximum of 1.39. The cumulative OR was 0.38 (95% CI 0.25–0.58; *p* < 0.0001), indicating a 62% lower risk of regional recurrence in patients undergoing neck treatment compared to patients who were directed to observation only (Figure 2). The funnel plot (Figure 3) did not show asymmetry, indicating the absence of publication biases and heterogeneity between the studies. Finally, the graphical representation of the risk of bias between the included studies is represented in Figure 4 and Figure 5.

## 4. Discussion

In the literature, the question of whether to perform an elective treatment on a clinical node-negative patient with SNCs remains open, and the present work may be considered as a natural prosecution of two other published meta-analyses that included only SCC [11] or SNUC [12]. Actually, squamous cell, adenocarcinomas, SNUC, and adenoid cystic carcinomas (ACC) are the most frequent histotypes that can yield nodal metastases in 28%, 25%, 12%. and 10% of cases, respectively [42]. A recent evaluation of the US National Cancer Database has estimated an overall risk of nodal disease for sinonasal cancer (all histotypes) of 13.2% at the moment of diagnosis, with sinus localization (vs. nasal cavity), black race, and uninsured patients as the principal risk factors [43]. By including over 1320 patients, we found a significantly higher nodal recurrence rate in patients who did not receive an upfront ENT (15.0%) versus patients who were treated with surgical dissection or irradiation (5.9%); while these findings might apparently favor ENT as the standard of treatment in cN0 SNCs, it remains unclear whether the added morbidity of ENT does actually outweigh the risk of having a regional failure [42,44].

An inherent issue of SNCs remains their biological heterogeneity that clinically translates into a different prognosis, radiosensitivity, and tendency to regional spread.

For example, a prospective propensity score matching study found no benefits in terms of event-free survival for END in cN0 ACCs; only a quarter of the cases were in the sinonasal cavities, but it should be noted that the vast majority of patients had postoperative RT, with fields probably involving at least the I levels [45]. Another study from the US found improved OS only for END performed in T3-T4 major salivary glands ACC; in this large dataset of over 2800 patients, the sinonasal cavity represented the second anatomical subsite in terms of frequency, but it was also the least likely to receive END and to harbor occult metastases [46]. Finally, in a recently published series of exclusively sinonasal ACC treated by endoscopic endonasal approaches, nodal recurrence occurred in only 1 out of 30 cases, and it was concomitant to distant metastases; again, adjuvant RT was delivered in 83% of patients but the fields were not specified [47].

For SNUC lesions, in the series from the Mayo Clinic, in those patients who received ENI, no regional recurrences were documented with a median follow-up time of 6.9 years; only one patient died of regional failure, but their initial neck status was not specified [48]. In a meta-analysis of SNUC patients, nodal metastases were present in 16% at diagnosis and, thus, the authors always advocated for the inclusion of the neck in the planning of irradiation fields [49].

Instead, for adenocarcinomas, a recent SEER analysis revealed no significant differences in terms of OS or DSS when comparing active surgical management to observation in the N0 neck (OS *p* = 0.83, DSS *p* = 0.82) [50]. Most notably, the presence of nodal metastases at diagnosis represented a negative prognostic factor at multivariate analysis.

Currently, the most recent NCCN guidelines recommend ENT only in patients with T3 to T4a tumors of the maxillary sinus [51]. The rationale for prophylactic neck treatment is based on the classical model proposed by Weiss et al. [52] more than twenty years ago; however, the cut-off of a preoperative risk of occult nodal disease of 20% was based only on squamous-cell carcinomas and whatever the anatomical subsite. Given the rarity of these malignant tumors, the majority of the included studies have a large time span, with the earliest cases dating back to the 1960s [32]. This feature obviously introduces an unavoidable bias in terms of correct pretreatment staging and, as already discussed in the work by Mirghani et al. [44], the highest rates of occult nodal disease were found in older publications. Whether this was related only to a late diagnosis with associated high T SNCs is not clear, given that, in recent years, delayed presentation of these tumors is still frequent, and T3–T4 cancers still represent the majority of cases [53].

The association between higher T stages and the risk of nodal occult disease should be always kept in mind; for instance, in a series of 299 patients with sinonasal malignancies, the higher cumulative incidence of regional metastases was significantly linked to dural, orbit, or infratemporal fossa involvement, i.e., T4 tumors by definition [9]. An unresolved question is whether there has been a reduction of late presentation at diagnosis for these particular tumors. Unfortunately, because of missing data, changes in the TNM staging system, and of the long timespan of the published series, this was basically never assessed, even in studies specifically addressing the temporal trends of SNCs’ epidemiology [6,54]. In the near future, it remains to be verified whether improvements in both radiological and endoscopic techniques would allow for a parallel change in this setting [55,56].

It must be remembered that the NCCN recommendations are not consistently followed at all institutions and that, for instance, due to the possible involvement of retropharyngeal nodes, END is not performed, not even for maxillary sinus cancer, where some authors may deem it necessary in order to isolate blood vessels for microvascular anastomosis after maxillectomy [57,58]. Interestingly, retropharyngeal lymph nodes were almost never mentioned (see Table 2) as sites of nodal failure, and this is despite their known prognostic impact [7]. Regional recurrences portend a significantly worse prognosis for SNCs [59], but a major obstacle to the standard implementation of ENI is represented by the fact that isolated regional failures are very rare and, therefore, there is a lack of perceived benefit [42]. Actually, patients who experienced nodal relapse frequently died because of subsequent distant metastasis or combined local disease progression [33,42,58,59].

Our work suffers from some of the well-known issues related to sinonasal malignant tumors: because of their rarity and of the heterogeneity of the many described histotypes, only limited and small studies are usually reported. Furthermore, there is always the risk that some advanced-stage squamous cell carcinomas of the maxillary sinus and of the nasal cavity could actually represent tumors of the oral cavity or the skin, respectively. Another inherent bias comes from the imaging techniques that were chosen to stage the pretreatment nodal status; as was reported in Table 1, some groups only relied on physical examination or simple x-rays, which have notoriously low diagnostic accuracy. In addition, it was not possible to analyze the specific cause of death because they were almost never reported, nor was it always clear whether successful salvage neck dissection could be more easily accomplished in the ENT or observation group. These factors make it very difficult to ascertain how regional relapses actually contribute to mortality, and we strongly hope that a future published series will not forget to report this information.

## 5. Conclusions

This is the first meta-analysis investigating the role of ENT in the management of clinically node-negative sinonasal carcinomas. Our results show that the “observation” approach on the neck portends a significantly higher regional recurrence rate, and they suggest that, despite continuous improvement in imaging techniques, nodal micrometastases can be present at the moment of diagnosis. In this regard, an elective dissection or irradiation should be taken into consideration in order to minimize the risk of recurrence in the neck and the need for salvage surgery. Specific studies are still needed, specifically in order to clarify whether a reduction in the nodal recurrence rate does actually imply a reduction in the disease-specific mortality rate.

## Figures and Tables

**Table 1:** A descriptive overview of the articles included in the meta-analysis. AC: adenocarcinoma; ACC: adenoid-cystic carcinoma; NR: not reported; SCC: squamous cell carcinoma; SNUC: sinonasal undifferentiated carcinoma; US: ultrasound; CT: computed tomography; MRI: magnetic resonance imaging; (C)RT: Radiotherapy +/− chemotherapy; OS: overall survival.

	No. of Patients (Sex)	Age (Years: Mean; Range)	Years	Final Histopathology	Subsite	T Stage	N Stage	N Evaluation	Treatment	Follow-Up Time (Months: Mean, Range)	OS
Al-Mamgani et al. (2013) [16]	21 (11 M—10 F)	52; 26–78	1996–2010	21 SNUC	5 maxillary, 16 ethmoidal	6 T3, 6 T4a, 9 T4b	19 N0, 2 N+	US, CT or MRI	7 (C)RT, 14 Surgery +(C)RT	54, 26–78	5-year OS 74%
Ang et al. (1992) [17]	45 (29 M—16 F)	58; 19–73	1969–1985	30 SCC, 9 AC, 5 ACC, 1 SNUC	45 nasal cavity	NR	44 N0, 1 N+	physical examination, plain X-rays, polytomograms (after 1975 CT)	18 (C)RT, 27 Surgery + (C)RT	132, 36–204	5-year OS 75%
Bhasker et al. (2017) [18]	16 (13 M—3 F)	47.5; 8–65	2004–2012	16 SNUC	7 nasal cavity, 5 maxillary, 3 ethmoidal, 1 sphenoidal	1 T3, 15 T4	13 N0, 3 N+	CT and/or MRI	1 Surgery, 10 (C)RT, 3 Surgery + (C)RT	10, 1–43	2-year OS 47%
Bristol et al. (2007) [19]	146 (86 M—60 F)	59; 26–90	1969–2002	89 SCC, 6 AC, 33 ACC, 11 SNUC, 7 Others	146 maxillary	22 T1-T2, 47 T3, 77 T4	126 N0, 20 N+	physical examination, plain X-rays, (after 1975 CT)	146 Surgery + (C)RT	46, 4–357	5-year OS 55%
Brown et al. (2013) [20]	18 (10 M—8 F)	62; 39–89	1992–2008	18 SCC	18 maxillary	2 T2, 2 T3, 14 T4a	17 N0, 1 N1	NR	3 Surgery, 15 Surgery + (C)RT	102, 33–206	5-year OS 37%
Castelnau-Marchand et al. (2016) [21]	104 (62 M—42 F)	64; 17–92	1998–2012	104 SCC	20 nasal cavity, 70 maxillary, 14 ethmoidal	8 T1, 9 T2, 18 T3, 69 T4	87 N0, 4 N1, 3 N2a, 6 N2b, 4 N2c	CT and/or MRI	14 Surgery, 30 (C)RT, 60 Surgery + (C)RT	54, NR	5-year OS 50%
Chen et al. (2008) [22]	21 (14 M—7 F)	47; 33–71	1990–2004	21 SNUC	11 nasal cavity, 5 maxillary, 5 ethmoidal	4 T3, 9 T4a, 8 T4b	19 N0, 2 N+	CT and MRI	4 (C)RT, 17 Surgery + (C)RT	58, 12–70	5-year OS 43%
Chobe et al. (1988) [23]	32 (NR)	NR; 43–77	1963–1984	32 SCC	32 nasal cavity	NR	32 N0	physical examination	32 (C)RT	NR	NR
Christopherson et al. (2014) [24]	23 (14 M—9 F)	56.5; 23–83	1992–2010	23 SNUC	NR	NR	18 N0, 5 N+	NR	8 (C)RT, 15 Surgery + (C)RT	36, 12–240	5-year OS 32%
Doescher et al. (2015) [25]	44 (33 M—11 F)	61; 37–84	1994–2013	44 SCC	NR	22 T1, 11 T2, 6 T3, 5 T4	42 N0, 2 N+	NR	30 Surgery, 1 (C)RT, 13 Surgery + (C)RT	84, NR	5-year OS 69%
Donhujsen et al. (2016) [26]	117 (NR)	NR	2000–2013	117 AC	NR	12 T1, 60 T2, 20 T3, 25 T4	117 N0	NR	36 Surgery, 4 (C)RT, 77 Surgery + (C)RT	60, NR	5-year OS 26%
Duru Birgi et al. (2015) [27]	43 (25 M—18 F)	67; 41–86	2007–2012	43 SCC	22 nasal cavity, 20 maxillary, 1 ethmoidal	6 T1, 6 T2, 2 T3, 23 T4a, 6 T4b	38 N0, 4 N1, 1 N2c	CT and/or MRI	18 (C)RT, 25 Surgery + (C)RT	NR	2-year OS 71%
Fornelli et al. (2000) [28]	32 (21 M—11 F)	65; NR	1976–1993	32 SCC	32 nasal cavity	NR	32 N0	NR	15 Surgery, 9 (C)RT, 8 Surgery + (C)RT	42, 9–156	5-year OS 50%
Hinerman et al. (2011) [29]	54 (34 M—20 F)	62; 36–79	1969–2006	54 SCC	54 maxillary	2 T2, 13 T3, 22 T4a, 17 T4b	45 N0, 5 N1, 1 N2a, 2 N2b, 1 N2c	NR	32 (C)RT, 22 Surgery + (C)RT	18, NR	5-year OS 41%
Jiang et al. (1991) [30]	73 (41 M—32 F)	53; 27–76	1969–1985	36 SCC, 6 AC, 20 ACC, 9 SNUC, 2 Others	73 maxillary	3 T1, 16 T2, 32 T3, 22 T4	67 N0, 4 N1, 2 N2	physical examination, plain X-rays, (after 1975 CT, occasionally MRI)	73 Surgery + (C)RT	83, 9–182	5-year OS 48%
Kim et al. (2004) [31]	8 (6 M—2 F)	48; 27–68	1995–2002	8 SNUC	NR	NR	8 N0	NR	1 Surgery, 3 (C)RT, 4 Surgery + (C)RT	NR	2-year OS 75%
Le et al. (2000) [32]	97 (67 M—30 F)	58; 20–85	1959–1996	58 SCC, 4 AC, 19 ACC, 16 SNUC	97 maxillary	8 T2, 36 T3, 53 T4	86 N0, 6 N1, 3 N2b, 2 N2c	physical examination (after 1977 CT, occasionally MRI)	36 (C)RT, 61 Surgery + (C)RT	78, 18–276	5-year OS 31%
Lee et al. (2018) [33]	124 (85 M—39 F)	57.5; 33–82	2000–2015	82 SCC, 5 AC, 23 ACC, 14 Others	109 maxillary, 13 ethmoidal, 2 sphenoidal	13 T2, 58 T3, 53 T4	124 N0	CT or MRI	26 (C)RT, 98 Surgery + (C)RT	54, 2–288	5-year OS 67%
Levendang et al. (1990) [34]	63 (57 M—6 F)	64; 33–84	1978–1988	63 SCC	63 nasal cavity	36 T1, 24 T2	59 N0, 2 N1, 2 N2a	NR	63 (C)RT	46, NR	5-year OS 65%
Lopez et al. (2015) [35]	17 (9 M—8 F)	53; 28–73	2001–2013	17 SNUC	17 ethmoidal	1 T3, 4 T4a, 12 T4b	15 N0, 2 N+	CT or MRI	3 (C)RT, 14 Surgery + (C)RT	48, 6–96	5-year OS 58%
McCollough et al. (1993) [36]	39 (23 M—16 F)	65; 40.84	1968–1988	39 SCC	39 nasal cavity	13 T1, 8 T2, 18 T4	37 N0, 1 N1, 1 N2b	NR	39 (C)RT	24, NR	5-year OS 75%
Mendenhall et al. (1987) [37]	22 (NR)	NR	1964–1984	22 SCC	22 nasal cavity	7 T1, 2 T2, 2 T3, 11 T4	20 N0, 2 N1	NR	22 (C)RT	90, NR	5-year OS 75%
Parsons et al. (1996) [38]	35 (NR)	NR	1964–1992	20 ACC, 15 Others	14 nasal cavity, 12 maxillary, 7 ethmoidal, 2 sphenoidal	4 T1-T2, 10 T3, 21 T4	35 N0	NR	18 (C)RT, 17 Surgery + (C)RT	NR	5-year OS 43%
Revenaugh et al. (2011) [39]	13 (7 M—6 F)	49; 16–78	2002–2009	13 SNUC	3 maxillary, 4 ethmoidal, 4 sphenoidal, 2 frontal	1 T1, 3 T4a, 9 T4b	12 N0, 1 N2c	NR	5 (C)RT, 8 Surgery + (C)RT	23, 3–62	2-year OS 80%
Snyers et al. (2009) [40]	98 (NR)	NR	1986–2006	55 SCC, 43 AC	32 nasal cavity, 22 maxillary, 22 ethmoidal	1 T1, 9 T2, 22 T3, 21 T4a, 23 T4b	73 N0, 1 N1, 2 N2b	physical examination (in some cases CT, MRI or US + FNAC)	NR	69, 3–253	5-year OS 35%
Tanzler et al. (2008) [41]	15 (10 M—5 F)	57; 23–82	1992–2005	15 SNUC	NR	8 T4a, 7 T4b	13 N0, 1 N1, 1 N2c	CT and/or MRI	1 Surgery, 5 (C)RT, 9 Surgery + (C)RT	30, 11–151	3-year OS 67%
**Total**	**1320** **(657 M—359 F—304 NR)**			**801 SCC, 190 AC, 120 ACC, 171 SNUC,** **38 Others**	**339 nasal cavity,** **639 maxillary, 102 ethmoidal,** **9 sphenoidal,** **2 frontal**	**305 T1-T2,** **280 T3,** **570 T4 (110 T4a, 91 T4b, 369 not specified)**	**1198 N0,** **31 N1,** **32 N2** **(6 N2a, 14 N2b, 10 N2c, 2 not specified)**		**101 Surgery,** **393 (C)RT,** **726 Surgery + (C)RT**		

**Table 2:** Regional recurrence in N0 patients according to the type of neck management. BSC: best supportive care; IRR: isolated regional recurrence; DIRR: dead from isolated regional recurrence; DLRD: dead from locoregional disease; DOD: died of disease; ND: neck dissection; PORT: post-operative radiotherapy; NR: not reported.

	No. Patients	Regional Recurrence	ENT Group				Observation Group			
			Recurrences	Levels	Salvage Neck Strategy	Survival	Recurrences	Levels	Salvage Neck Strategy	Survival
Al-Mamgani et al. [16]	19	2	0/8	-	-	-	2/11 (2 IRR)	NR	ND and PORT	no DOD
Ang et al. [17]	44	2	0/9	-	-	-	2/35 (2 IRR)	Subdigastrics	ND and PORT	no DOD
Bhasker et al. [18]	13	1	0/5	-	-	-	1/8 (1 IRR)	NR	BSC	1 DIRR
Bristol et al. [19]	81	16	3/45 (0 IRR)	II ipsilateral	ND and PORT	no DOD	13/36 (8 IRR)	7 II ipsilateral, 4 Ib ipsilateral, 1 II bilateral, 1 II contralateral	NR	NR
Brown et al. [20]	17	2	2/16 (1 IRR)	ipsilateral	ND	1 DLRD, 1 DIRR	0/1	-	-	-
Castelnau-Marchand et al. [21]	87	8	5/55 (0 IRR)	Ib-II-III + intraparotid	NR		3/32 (2 IRR)	Ib-II-III + intraparotid	NR	NR
Chen et al. [22]	19	2	1/15 (0 IRR)	NR	NR	1 DLRD	1/4 (0 IRR)	NR	NR	1 DLRD
Chobe et al. [23]	32	4	0/14	-	-	-	4/18 (2 IRR)	NR	2 ND and PORT	2 DLRD
Christopherson et al. [24]	18	3	4/13 (NR)	NR	NR	NR	2/5 (NR)	NR	NR	NR
Doescher et al. [25]	42	2	0/16	-	-	-	2/26 (NR)	NR	NR	NR
Donhujsen et al. [26]	117	6	0/15	-	-	-	6/102 (3 IRR)	NR	NR	NR
Duru Birgi et al. [27]	38	4	0/11	-	-	-	4/27 (3 IRR)	1 II, 2 I, 1 facial	1 ND and PORT	3 DIRR
Fornelli et al. [28]	32	13	0/6	-	-	-	13/26 (NR)	most frequently level I	2 ND and PORT	11 DOD
Hinerman et al. [29]	45	3	1/23 (1 IRR)	II contralateral	NR	NR	2/22 (2 IRR)	1 I, 1 II	NR	NR
Jiang et al. [30]	67	11	0/17	-	-	-	11/50 (9 IRR)	3 Ib ipsilateral, 1 Ib bilateral, 7 subdigastric ipsilateral	2 BSC, 4 RT, 5 ND and PORT	7 DOD (5 of IRR group)
Kim et al. [31]	8	3	0/3	-	-	-	3/5 (3 IRR)	NR	NR	NR
Le et al. [32]	86	9	2/25 (NR)	I-II	NR	NR	7/61 (NR)	I-II	NR	NR
Lee et al. [33]	124	21	7/40 (NR)	5 in treated neck, 2 contralateral	NR	NR	14/84 (NR)	7 I ipsilateral, 6 II ipsilateral, 2 III ipsilateral, 1 IV ipsilateral, 1 I contralateral, 1 II contralateral, 1 I bilateral, 1 II III IV bilateral, 1 IV bilateral, 1 nr ipsilateral	NR	NR
Levendang et al. [34]	59	4	2/25 (NR)	NR	NR	NR	2/34 (NR)	NR	NR	NR
Lopez et al. [35]	15	3	0/10	-	-	-	3/5 (NR)	NR	1 ND and PORT	1 DIRR, 1 DLRD
McCollough et al. [36]	37	4	0/5	-	-	-	4/32 (NR)	1 jugulodigastric ipsilateral, 2 submaxillary, 1 facial	ND +/- PORT	no DOD
Mendenhall et al. [37]	20	2	0/3	-	-	-	2/17 (NR)	Ib	ND	no DOD
Parsons et al. [38]	35	1	0/15	-	-	-	1/20 (NR)	NR	NR	NR
Revenaugh et al. [39]	12	2	0/3	-	-	-	1/9 (NR)	II ipsilateral	ND	no DOD
Snyers et al. [40]	98	10	0/3	-	-	-	10/95 (NR)	NR	NR	NR
Tanzler et al. [41]	13	2	0/7	-	-	-	2/6 (NR)	NR	NR	1 DIRR, 1 DLRD
**Total**	**1178**	**140**	**24/407**				**115/771**			

## Data Availability

Data can be obtained upon reasonable request to the Corresponding Author.
